# eMental Health Literacy and the Relationship to Barriers to Mental Health Care

**DOI:** 10.1093/geroni/igaa057.978

**Published:** 2020-12-16

**Authors:** Eve Root, Grace Caskie

**Affiliations:** Lehigh University, Bethlehem, Pennsylvania, United States

## Abstract

According to the American Psychological Association (2017), one in four individuals who are 65 years and older experience a mental health problem; however, many older adults do not receive the services they need and deserve (Karlin, 2008). The current study utilizes a new concept similar to eHealth Literacy called eMental Health Literacy, defined as the degree to which individuals obtain, process, and understand basic mental health information and services needed to aid their recognition, management, or prevention of mental health issues. The relationship of eMental Health Literacy to perceived barriers to receiving mental health services was examined in a sample of middle-aged and older adults. We hypothesized that higher eMental Health Literacy would predict fewer reported barriers to mental health services. A sample of 243 participants (M=63.33, range=55-80 years) were recruited online through Amazon Mechanical Turk to complete measures assessing eMental Health Literacy (eMHEALS) and mental health barriers (BMHSSS-R). After adding two correlated errors, a structural equation model specifying eMHEALS as a predictor of extrinsic and intrinsic barriers to mental health services achieved good fit (χ2(60)=170.014, p<.001, SRMR=.068, CFI=.944, GFI=.901, TLI=.927, RMSEA=.087). All indicators were significantly related to their latent construct (p<.001). The results indicate higher eMental Health Literacy significantly predicted fewer reported intrinsic and fewer extrinsic barriers to mental health services. These relationships were statistically significant even when examined across differing socioeconomic status and age. These findings indicate eMental Health Literacy may have significant impact on the way individuals in later life navigate through the mental healthcare system.

